# The cost of cancer care: how far would you go for a trial?

**DOI:** 10.1007/s11845-021-02915-6

**Published:** 2022-01-17

**Authors:** Orla M. Fitzpatrick, Catherine Murphy, Erica Duignan, Keith Egan, Bryan T. Hennessy, Liam Grogan, Adrian Murphy, Oscar S. Breathnach, Jarushka Naidoo, Patrick G. Morris

**Affiliations:** Cancer Clinical Trials and Research Unit, Medical Oncology Department, Beaumont RCSI Cancer Centre, Beaumont Hospital, Dublin, Ireland

**Keywords:** Clinical trials, Distance, Expense, Oncology

## Abstract

**Background:**

Clinical trials are often considered the gold standard in cancer care. However, patients face barriers in trial participation including distances to cancer centres and personal costs including changing employment status, cost of medications, inpatient admissions, and parking tariffs.

**Aim:**

Our aim was to compare the distances patients travelled for clinical trials compared to those receiving standard systemic anticancer therapy (SACT). We also investigated the additional costs associated with this.

**Methods:**

This was a retrospective review of electronic patient medical records. The distance from the patients’ home address to Beaumont was calculated as a one-way journey in kilometres. Patients attending for clinical trials were compared to those receiving standard of care SACT.

**Results:**

A total of 271 patients receiving standard SACT over a 5-day period and 111 patients enrolled on 24 clinical trials were included. The median one-way distance travelled by patients enrolled in clinical trials was 41.4 km, compared to 14 km in those patients’ receiving standard of care SACT. The median estimated cost was €13 vs €4.20 for those enrolled on clinical trials compared to those receiving standard of care treatment, respectively.

**Conclusion:**

Patients enrolled on clinical trials often travel more than twice as far to receive their anti-cancer treatment compared to those receiving standard of care SACT and incur an increased cost of travel expenses.

## Introduction

Clinical trials offer patients undergoing systemic anti-cancer therapy (SACT) the opportunity to get access to novel approaches and potentially better treatment. Trials are often considered the gold standard and a marker of high-quality clinical care. However, the conduct of clinical trials is complex and requires specialist infrastructure, which is usually provided in cancer centres. Internationally, many patients must travel long distances to attend cancer centres in order to participate in clinical trials [1]. In Ireland, there is increasing centralisation of some services as part of the hospital group system as well as strengthening links with universities [2]. The new grant model from the Health Research Board (HRB), due to commence in 2022, has identified lead centres within clusters for the conduct of cancer clinical trials.

A barrier to cancer care delivery is the distance that patients travel for both cancer diagnosis and access to standard of care SACT [3]. Poor physical wellbeing has been related to remoteness from the treatment centre and thus influences the quality of life and patient outcomes, highlighting that clinicians should begin to take geographical location from the treating hospital into account when planning for treatment [4, 5]. Increased travel time has also been shown to negatively impact cancer staging at diagnosis [5]. In Ireland, the majority of patients use their own car to attend the hospital, and the median monthly expenditure for this was €31 [6]. Although there are two options for reimbursement of some travel costs in Ireland, many patients do not qualify. The first is the Travel2Care allowance which is available to those with a genuine financial need, living further than 50 km in one direction from the 13 hospitals eligible for this allowance, and the second is a Volunteer Driver Service which relies on volunteers to drive patients to and from 21 eligible hospitals. Patients enrolled on clinical trials have an increased burden of hospital appointments, and while some trials can provide limited remuneration, the travel burden is less for those receiving standard of care and may influence patients’ decisions to enrol [1, 7–9].

We aimed to assess the distance travelled by patients in Ireland from their home to their cancer centre at Beaumont Hospital in Dublin. Our primary aim was to compare the distance that patients travel when they are receiving standard of care treatment to those enrolled on a clinical trial and subsequently to compare the cost of these journeys. Some clinical trials are particularly specialised, and patients might travel from across the county based on their tumour type and possible access to novel therapy. A retrospective study was conducted to identify patterns of patient travel for cancer clinical trials and to identify and cost barriers to participation.

## Methods

### Patients

This was a retrospective review of electronic medical records. Data on patient demographics were collected. Patients attending for the standard of care treatment over a 5-day period were included in the standard arm. These data were compared with data from patients registered on 24 clinical trials conducted over a 3-year period. Patients were grouped based on their cancer type into the following: central nervous system (CNS), genito-urinary, gastrointestinal, lung, breast, and other cancer types.

### Distance and costing

The distance from the patients’ home address to Beaumont was calculated as a one-way journey in kilometres. The cost of the journeys was estimated based on a median-priced new car in Ireland which was €33,287 in 2020 [10]. This pricing was based on motor insurance company data. A 1.5L Opel Astra was used to estimate the cost of these journeys. The Michelin Route Planner calculated the cost of the journey via the shortest possible route, and the cost of tolls was included in the cost of the journey [11].

## Results

A total of 271 patients receiving standard SACT over a 5-day period and 111 patients enrolled on 24 clinical trials were included. Patient demographics are summarised in Table 1. The median age was 58 and 59 years for patients on clinical trials versus standard SACT. The majority of patients enrolled on clinical trials were women (67% female vs 33% male) compared to those receiving standard of care SACT (51% female vs 49% male).

**Table 1:** Patient demographics of patients enrolled on clinical trials and those receiving standard of care treatment

	**Clinical trial** ***N* (%)**	**Standard of care** ***N* (%)**
**Total no. of patients**	111	271
**Median age**	58	59
**Age range**	30–76	18–86
**Gender**		
** Male**	37 (33)	133 (49)
** Female**	74 (67)	138 (51)
**Disease stage**		
** Early**	57 (51)	99 (37)
** Advanced**	54 (49)	172 (63)
**Disease type**		
** Breast**	51 (46)	72 (27)
** Gastrointestinal**	20 (18)	61 (23)
** Central nervous system**	13 (12)	31 (11)
** Genitourinary**	6 (5)	48 (18)
** Lung**	6 (5)	29 (11)
** Others**	15 (14)	30 (10)

**Fig. 1:**
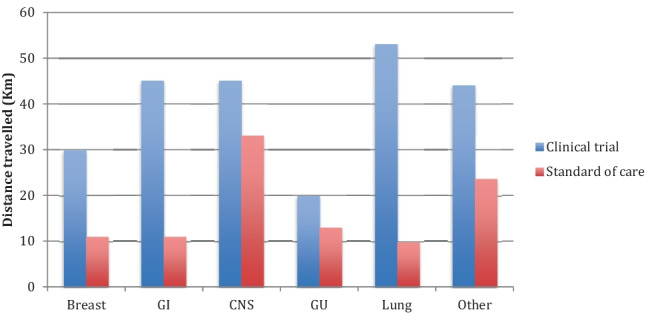
Distance travelled by patients with each tumour type: breast, gastrointestinal (GI), central nervous system (CNS), genitourinary (GU), lung, and other cancer types

The median one-way distance travelled by patients enrolled in clinical trials was 41.4 km, compared to 14 km in those patients receiving standard of care SACT. The distance discrepancy between clinical trial patients and patients receiving standard of care SACT was seen throughout all cancer groups and is shown in Fig. 1; breast cancer patients travelled a median distance of 29.8 km vs 11 km, patients with GI cancer travelled 45 km vs 11 km, patients with CNS cancer travelled 45 km vs 33 km, patients with genitourinary cancer travelled 19.8 km vs 13 km, patients with lung cancer travelled 53 km vs 9.8 km, and patients with other cancer types travelled a median distance of 44 km vs 23.5 km.

**Fig. 2:**
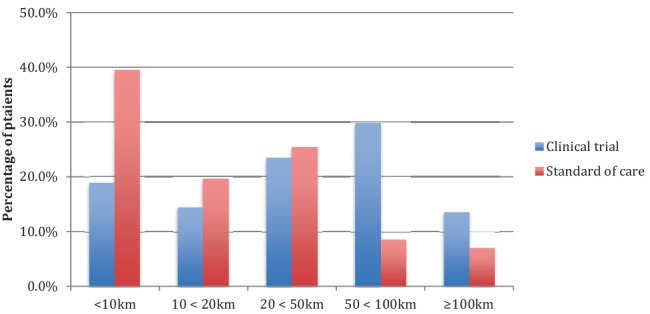
Proximity of patients enrolled on clinical trials and receiving standard of care treatment to Beaumont Hospital

**Fig. 3:**
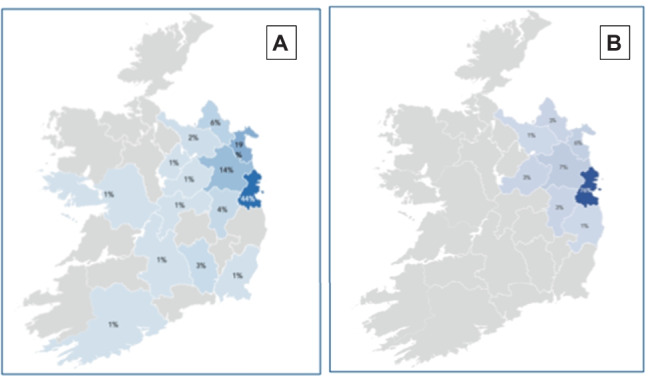
Geographical distribution of patients enrolled on clinical trials (**A**) and those receiving standard of care treatment (**B**)

**Fig. 4:**
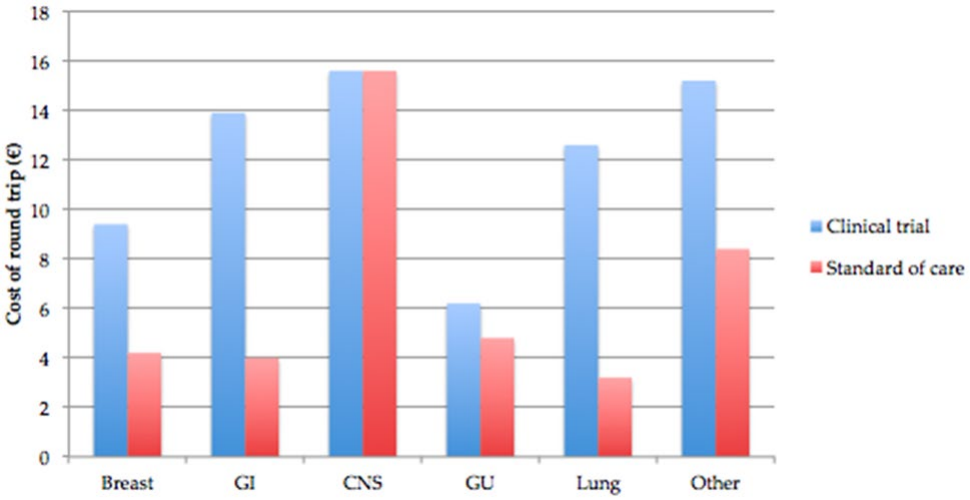
Median cost of journeys by tumour type: breast, gastrointestinal (GI), central nervous system (CNS), genitourinary (GU), lung, and other cancer types

The highest proportion of patients enrolled on clinical trials lived between 50 and 100 km away from Beaumont Hospital (29.7%, 33/111), compared to patients receiving standard of care SACT where most patients lived within a 10-km distance (39.4%, 107/271), shown in Fig. 2. This data is shown by geographical distribution in Fig. 3. Mirroring the distance discrepancy between clinical trials and standard of care journeys, the cost of journeys is estimated to be higher for those enrolled on clinical trials which is shown in Fig. 4 (€13 vs €4.20), except for those patients with CNS malignancies where the cost was estimated to be the same at €15.60.

## Discussion

In this retrospective trial, patients enrolled on clinical trials travelled on average more than twice as far to receive treatment compared to those receiving standard of care SACT and incur an increased cost of travel expenses. This study included a large number of cancer clinical trials over a prolonged period, covering multiple types of cancer, and was compared to a cohort of patients receiving standard of care SACT with equally varying types of cancer. Beaumont Hospital is one of the main neuro-oncology centres in Ireland, and patients with CNS malignancy, whether enrolled on a clinical trial or receiving standard of care SACT, may travel further for their treatment, which we demonstrated by showing the estimated journey cost was similar for both cohorts.

Patients receiving standard of care SACT still incur a cost, albeit reduced, and the current support services are limited in Ireland for those under financial strain. Overall, personal costs are multifactorial and can be grouped into 3 main areas: (1) medical expenses including prescriptions for supportive medications, inpatient admissions, physiotherapy, occupational therapy, private health insurance, and increased frequency of GP visits; (2) non-medical costs including travel expenses, changing employment status, and parking tariffs; and (3) miscellaneous purchases including wigs, specialist equipment, specific support clothing, changing childcare costs, and counselling. These headings are not considered to be exhaustive, and many patients incur additional costs [12]. In Ireland, this cost has been conservatively estimated to be €291 [13] per month by the Irish Cancer Society, but can rise to over €1000 per month in some cases [13]. These are considered to be hidden costs, many being non-reimbursable, which can accumulate from the time of diagnosis through treatment and ultimately influence patients’ decisions to continue with treatment [14, 15]. These costs, combined with the fact that up to 67% of patients report a reduction in their income as a direct result of their cancer diagnosis, can result in distress to patients [12, 16, 17].

Reduced socioeconomic status has been associated with reduced engagement with cancer services and lower rates of clinical trial participation as well as increased distress and worsened quality of life [18–21]. These data support the need to improve the current financial support available to patients with cancer, to not affect engagement with cancer services and enrolment in clinical trials [22–24]. An analysis of Dublin City socioeconomic profile conducted by Dublin City Council showed that the area around North Dublin city, including the Beaumont Hospital catchment area, has the lowest proportion of people who attained a third level education and that neighbourhoods located in this area have the highest levels of disadvantage, alongside certain areas in West Dublin [25]. In comparison to areas in South Dublin city, there is a lower employment rate [25]. These factors have been independently shown to have a negative impact on engagement with healthcare services and clinical trials [8, 26–28].

Location has also been known to affect accrual to clinical trials, both with regard to the time taken to travel and incurred expenses from travel [29, 30]. Currently, there are 18 cancer trial research units in Ireland, of which 8 are in Dublin. This leaves large regions of the country with longer travel times to these centres, with only 1 centre located in the midlands. This affects not only accrual, but also retention of these patients on clinical trials [7].

Since March 2020, the COVID-19 pandemic in Ireland has been disruptive to the delivery of cancer care [31]. Initially, when the effects of COVID-19 on those undergoing SACT were unknown, many non-urgent treatments and investigations were delayed [32]. Focus shifted towards managing COVID-19 in hospitals and away from clinical trials. COVID-19 has heightened the disparities between socioeconomic status and access to cancer care and emphasises the need for access to clinical trials locally, to prevent patients from travelling to multiple sites for their care [32, 33]. COVID-19 has also been shown to affect enrolment, and this may be related to the increased burden of hospital visits required during clinical trials [34]. This not only highlights the need for access to clinical trials on a local level, but also the need to streamline the enrolment and retainment process [31, 34, 35].

The costing in this study was based on all patients driving a median-priced car in Ireland, based on data from motor insurance companies [36]. This provided an estimated rather than an individualised cost; however, discrepancies of fuel efficiency can vary between cars, and some patients may have relied on family, friends, or volunteers to drive them to appointments and hence incur no personal cost. This costing was also based on the assumption that all patients travelled the shortest possible distance to reach Beaumont Hospital, and anecdotally some patients report travelling further to avoid toll costs, resulting in an overall cheaper journey. These data do not account for patients enrolled on travel allowance schemes as this is coordinated through the Irish Cancer Society.

In conclusion, this research has identified that many patients travel long distances to enrol on cancer clinical trials. A holistic approach to cancer care delivery is needed, which encompasses both socioeconomic factors, available financial support, and proximity to the cancer centre.
